# What is driving global obesity trends? Globalization or “modernization”?

**DOI:** 10.1186/s12992-019-0457-y

**Published:** 2019-04-27

**Authors:** Ashley Fox, Wenhui Feng, Victor Asal

**Affiliations:** Rockefeller College of Public Affairs and Policy, University at Albany, State University of New York, Albany, USA

**Keywords:** Obesity, Body mass index, Globalization, Women’s empowerment, Nutrition transition

## Abstract

**Background:**

Worldwide obesity has more than doubled since 1980. Researchers have attributed rising obesity rates to factors related to globalization processes, which are believed to contribute to obesity by flooding low-income country markets with inexpensive but obesogenic foods and diffusing Western-style fast food outlets (dependency/world systems theory). However, alternative explanations include domestic factors such as increases in unhealthy food consumption in response to rising income and higher women’s labor force participation as countries develop economically (“modernization” theory). To what extent are processes of globalization driving rising global overweight/obesity rates versus domestic economic and social development processes? This study evaluates the influence of economic globalization versus economic development and associated processes on global weight gain.

**Results:**

Using two-way fixed-effects OLS regression with a panel dataset of mean body weight for 190-countries over a 30-year period (1980–2008), we find that domestic factors associated with “modernization” including increasing GDP per capita, urbanization and women’s empowerment were associated with increases in mean BMI over time. There was also evidence of a curvilinear relationship between GDP per capita and BMI: among low income countries, economic growth predicted increases in BMI whereas among high-income countries, higher GDP predicted lower BMI. By contrast, economic globalization (dependency/world systems theory) did not significantly predict increases in mean BMI and cultural globalization had mixed effects. These results were robust to different model specifications, imputation approaches and variable transformations.

**Discussion:**

Global increases in overweight/obesity appear to be driven more by domestic processes including economic development, urbanization and women’s empowerment, and are less clearly negatively impacted by external globalization processes suggesting that the harms to health from global trade regimes may be overstated.

**Electronic supplementary material:**

The online version of this article (10.1186/s12992-019-0457-y) contains supplementary material, which is available to authorized users.

## Background

Worldwide obesity has more than doubled since 1980 and most of the world’s population now live in countries where overweight and obesity kills more people than underweight [45, 75]. Research on the relationship between economic development processes and health has identified several competing structural explanations for rising body mass index (BMI) including globalization processes, economic development and women’s changing role in society that likely affect changes in underlying behavioral mechanisms. Previous research has not systematically tested these different explanations for the global rise in obesity.

A growing body of literature has drawn attention to the ways that economic globalization, particularly trade liberalization, has contributed to global weight gain by facilitating the diffusion of obesogenic products such as sugar-sweetened beverages and packaged foods to low and middle income countries (LMICs) [2, 10, 17, 18, 28, 29, 37, 46, 64, 65, 72]. In addition, other elements of globalization apart from economic globalization may contribute to rising obesity rates globally. Cultural globalization, or “Westernization,” may encourage the consumption of fast foods like McDonalds to appear more “modern” [57, 73]. This may be less due to economics and more due to the cultural appeal of Western lifestyles, which can contribute to obesity as people abandon local cuisines for Western-influenced diets [49]. These “world systems theory” accounts place the explanation for countries’ widening waistlines largely on factors external to the country – i.e., international trade regimes that have allowed the entry of transnational food corporations into emerging economies driving increased consumption of unhealthy foods and ideational lifestyle diffusion [58].

However, an alternative explanation for the global rise in obesity is that countries are experiencing domestic “nutrition transitions,” or the shift from a primarily plant-based diet to a meat and processed food diet associated with weight gain and chronic illnesses [50, 52]. Even in the absence of increased exposure to global markets or images of the Western consumerism, burgeoning middle classes in countries may increase demand for a richer diet and prepared foods, including increased consumption of potentially unhealthy local foodstuffs. Modernization is also believed to set in motion a set of additional normatively positive developments that may also improve health in LMICs including urbanization, women’s rights and democratization [55]. In this “modernization theory” view of obesity, development inexorably leads to health transitions, including the rise of unhealthy lifestyles as people have more disposable income [4, 22].

However, development scholars have debated over whether modernization is a linear process, giving rise to immediate health improvements, or whether it forms more of a curvilinear, inverted-U shape relationship whereby the overall burden of disease may increase before declining owing to a double-disease burden [53, 54]. Moreover, technological diffusion facilitated by globalization may also serve to reduce disease burden or compress health transitions as health innovations may be more rapidly transferred and scaled-up in LMICs [53]. Thus, globalization may not be primarily a force for harm, foisting insalubrious products and habits on LMICs, but can also serve to transfer knowledge and leapfrog stages in development.

If modernization theory is correct, a linear relationship between GDP growth per capita and BMI should be observed whereas if dependency theory is correct, greater integration into the global economy and Western culture should contribute to more obesity. However, it is also possible that the relationship between economic development and weight is not linear and that at low levels of development weight increases rapidly but then levels off with higher levels of development.

**Fig. 1:**
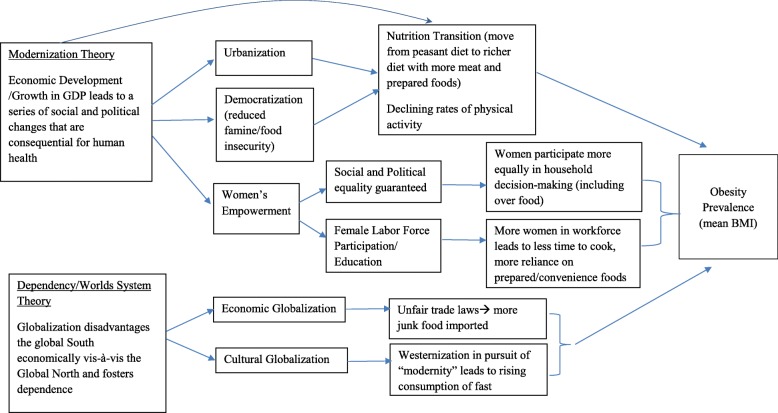
Theoretical Framework: How different Variables might Affect Men’s and Women’s Obesity

Though several studies have recently examined cross-national determinants of overweight/obesity in relation to globalization processes, previous studies have not attempted to explicitly test competing theories regarding the relationship between economic development and health across a full set of countries over a long time frame [8, 10, 11, 24, 35]. We test these different theories for the rise of global obesity rates using a longitudinal dataset of mean body mass index from the Global Burden of Metabolic Risk Factors of Chronic Diseases study and data from the Quality of Government dataset (QoG) that covers 190 countries from 1980 to 2008 (see Conceptual Model, Fig. 1). We improve on previous studies methodologically by using multiple imputation for missing values to ensure a balanced panel for all countries.

### Economic development, modernization theory and health

At low levels of development, health is generally thought to improve linearly with wealth [53, 54, 74]. In other words, as countries grow wealthier, health should improve in tandem. Proponents of this classical version of modernization theory suggest that economic growth and development should set in motion a series of normatively positive economic, social, cultural and political changes that would, interacting synergistically with one another, produce a “virtuous circle” of increasing living standards, social mobilization, democratization [36, 59] and improved health and human well-being [21]. Building on assumptions underlying modernization theory, classical demographic theory predicts a linear, secular decline in disease risk as wealth increases generating an epidemiologic transition from a high birth, high mortality dynamic driven by infectious diseases to a low-birth, low-mortality dynamic with death stemming largely from chronic illnesses [47]. As it pertains to nutrition and weight gain, classic works by Fogel [20] and McKeown [42] suggest that nutritional improvements (improved diet and synergies with infectious diseases) played a primary role in mortality reductions over the nineteenth century. Moreover, Fogel [21] suggests that improved nutrition has also contributed to improvements in human capital, which has served as a primary force promoting economic growth in the long term. However, while undernutrition may harm health, and adequate nutrition may be necessary for growth and reduced infectious-disease related mortality, recent attention has turned towards the role of overnutrition in contributing to the growing burden of chronic diseases in LMICs [65]. This conversion from a primarily plant-based diet to a meat and processed food diet associated with weight gain and chronic illnesses is referred to as the “nutrition transition” [51]. The global nutrition transition is believed to be contributing to rising obesity rates globally as well as increases in rates of chronic illness for which LMICs are believed to be unprepared [56].

Modernization theory might also predict BMIs to increase through the additional mechanisms of urbanization, women’s empowerment, and democratization. According to modernization theory, economic development is believed to unleash a series of normatively positive developments [36, 59] including industrialization, urbanization, increasing education levels, social mobilization, and the emergence of civil society- a progressive series of social change that ultimately culminate in democratization [55]. Of these processes, urbanization has previously been examined and found to be associated with rising obesity levels [7, 50, 51, 63]. People living in urban areas are believed to consume diets distinctly different from those of their rural counterparts and the general shifts in their diets enhance energy and fat density of foods consumed and may affect patterns of physical labor and activity [51].

Democratization might also have an impact on obesity, though it is not clear what the direction of the relationship might be. On the one hand, previous research has found that democracy reduces famines by ensuring government accountability and responsiveness [61]. Improved food security and nutritional status of the population might lead to higher BMIs. Countries that are democratic might adopt more consumer protection and regulatory policies that could also shield the public from obesogenic foods. On the other hand, there is little evidence presently that democracies have been more effective at protecting the public and citizens may not support against anti-obesity policies that are viewed as paternalistic.

Economic development may also contribute to changes in women’s role in society through changes in social relationships and family structure due to urbanization, labor force growth in industry and service [48]. However, the exact nature of the relationship between modernization and the development of women’s rights remains contested and others stress the importance of reducing patriarchal systems of oppression regardless of level of economic development [14]. Previous research has linked women’s labor force participation to rising obesity rates [1, 3, 5, 26]. Though the mechanisms are not well understood [5], working women may have less time to prepare healthy meals and may rely more on prepared foods [3]. Women that are more empowered socially and legally may also experience less constrained gender roles that tie them to traditional homemaking tasks including cooking.

However, other research has pointed to evidence of non-linearities in the relationship between wealth and health. While proponents of classical modernization theory view each of these processes as advancing in a linear fashion, critics of classical modernization theory have asserted that modernization is not in fact a linearly progressing, peaceful process, but rather one that results in social upheaval as a loosening of social controls with the decline of religious and traditional sources of meaning creates moral and normative vacuums [32, 60]. Adherents to this revised version of modernization theory, while still viewing development as unfolding in a relatively evolutionary and teleological manner, regard the process of economic development not as one unleashing a “virtuous circle,” but instead a “vicious circle” of rising expectations coupled with the inability of the state to respond to the growing demands of an increasingly engaged but thwarted populace. For instance, Szreter [70] has observed that rapid economic growth may actually cause health to get worse before it gets better generating more of an inverted-U shape relationship between development and disease burden. He attributes this to what he calls the “four D’s”- “disruption” of traditional ways of doing things, increasing relative “deprivation” followed by increases in “disease” and “death.”

In a similar vein to Szreter’s inverted U-shape relationship between development and health improvements, but operating at the individual level, the literature on the social determinants of health makes a distinction between diseases associated with absolute poverty and diseases that tend to be associated with relative poverty (e.g., [38, 39]). Examples of diseases associated with absolute poverty include malnutrition, diarrheal disease, and what are now considered to be neglected “tropical” diseases. These diseases are believed to be subject to a threshold effect such that once an individual is no longer exposed to the conditions that give rise to these diseases (i.e., vectors and disease hosts associated with a lack of sanitation, potable water, adequate nutrition, etc.), these diseases tend to decline on their own [38]. These diseases of poverty do not form social gradients and should in theory decline linearly with economic development.

By contrast, diseases of affluence associated with relative poverty should increase with development and then level off. Present day middle-income countries are beginning to experience what has come to be known as the “double disease burden”- or the coexistence of undernutrition and overnutrition-related non-communicable chronic diseases [67]. Risk factors associated with the lifestyles of a growing “leisure class” including changes in diet (the nutrition transition), smoking, and exercise that initially accrued to the rich in now developed countries are only just now emerging in the burgeoning middle classes of developing countries, whereas in rich countries the gradients in these lifestyles reversed decades ago [76]. Thus, middle income countries should have the highest overall burden of disease and one would expect obesity rates to be the highest in middle income countries that are presently in the midst of the nutrition transition.

### Globalization, dependency and health

Modernization theory and health transitions theories grounded in this view have been criticized for their teleological assumptions and linear world view as well as being undermined by a growing set of examples of countries that have not advanced on such a path. These explanations are further viewed as putting too much agency in the hands of countries and paying too little attention to dynamics in the international economy that function to perpetuate underdevelopment in low income countries. In contrast with modernization theory, dependency and world systems theories comprise a class of “radical” development theories, which suggest that poverty and underdevelopment is not so much the product of countries’ own making, but rather is largely the product of external exploitive factors acting upon countries [6, 16]. Though a full review of the literature on these theories is beyond the scope of this manuscript, according to this class of theories, the developing world was and continues to be dominated economically as well as politically by external centers of power. Debt, trade, and foreign investments pose negative effects on their populations’ health in this view.

While globalization and dependency/world system theories are arguably distinct, we, along with others, view the critiques of globalization as they pertain to health in LMICs as similar to criticisms advanced in the literature on dependency/world systems theory [27, 30, 34, 41]. Critics of globalization’s effects on health, for instance, have pointed to the role of “Big Food” in diffusing obesogenic food products globally. According to this view, the saturation of markets in developed countries, and the fact that people spend 20% of their income on average on food globally, has stimulated Big Food to seek global expansion [66]. Multinational food and beverage companies with concentrated market power have gained rapid entry into markets in low- and middle-income countries (LMICs) as a result of mass-marketing campaigns and foreign investment, principally through takeovers of domestic food companies. Three-fourths of world food sales involve processed foods, for which the largest manufacturers hold over a third of the global market [66]. In this world systems view, the flooding of markets in LMICs with highly processed, low-quality food stuffs is what can primarily account for recent increases in obesity rates in LMICs rather than domestic economic development processes per se.

A related explanation relies more heavily on the cultural appeal of Western lifestyles. As mass marketing diffuses Western processed foodstuffs including McDonalds, coco-cola and prepared foods, residents of LMICs begin shifting from traditional, local foodstuffs to a less healthy, stylized Western diet high in fat and sugar and low in nutrients. Through the spread of ideas, information, and images glorifying certain eating and leisure time activities, the public in LMICs may be influenced to adopt obesogenic behaviors. In both cases, increases in global BMI are viewed as resulting from international markets and diffusion processes and are less influenced by domestic social changes associated with economic development.

To test these different theories for the global rise in obesity, we assemble a longitudinal dataset comprising measures of mean body weight, economic and cultural globalization, GDP per capita, women’s empowerment and democracy for 190 countries over a 30-year period (1980–2008).

### Existing cross-national evidence on globalization, development and obesity

Several recent studies have examined cross-national determinants of overweight/obesity in relation to globalization processes [8, 10, 11, 24, 35]. Two studies, De Vogli et al. [10] and de Soysa and de Soysa [11], come closest to the present analysis employing cross-national time series analysis.

Drawing on a sample of 127 countries over the period 1980–2008, De Vogli et al. [10] et al. analyze the effects of economic globalization on BMI using time-series cross-section analysis. They find that economic globalization predicts increases in BMI with modest effect sizes (coef. = 0.008, *p* < 0.05) after adjusting for GDP per capita, which is also found to positively predict BMI. However, they employ few controls (only urbanization, proportion living in poverty and GDP per capita) and their sample excludes countries with missing data reducing country variability and sample size. They also do not disaggregate between male and female BMI.

de Soysa and de Soysa [11] examine the relationship between measures of economic and social globalization on childhood BMI measures (age 2–19) over the period 1990–2012 using cross-national time-series analysis with fixed effects across a sample of between 120 and 180 countries depending on the model. In contrast with De Vogli et al. [10] they find a *negative* relationship between economic globalization and childhood BMI and they find no relationship between social globalization and childhood BMI. They further find that several component parts of economic globalization such as trade openness, FDI flows, and an index of economic freedom reduce weight gain and obesity among children and youth leading them to conclude that “local-level factors possibly matter much more than do global-level factors for explaining why some people remain thin and others put on weight” [11]. While their focus on childhood obesity is a strength as this is the most likely age cohort to have been affected by the past three decades of globalization and associated lifestyle changes, their focus is more restricted as it does not encompass later life obesity rates, which would more plausibly be linked rising rates of chronic disease in older adults. The time frame and country sample is also more restricted. Both studies employ BMI data from the Global Burden of Metabolic Risk Factors Study and use the KOF globalization index as a metric for economic globalization.

Lawson et al. [35] also use a cross-national sample of 135 countries between 2000 and 2009 to examine the relationship between a measure of “economic freedom” and adult BMI. Economic Freedom of the World (EFW) index captures nations’ degree of reduced taxation, sounder property rights, stable money, freer trade, and more limited regulations score higher on this index. They find that economic freedom is associated with modestly higher BMIs for men (but not women) in developing nations. As with the previous studies, their country sample and time frame are more limited.

Finally, one other study has drawn on samples of individual-level data to examine the relationship between adult obesity (measured at the individual level) and macro-level measures of economic social and political globalization. Covering 56 countries, Goryakin et al. [24] find that economic globalization reduces obesity among adult women while social and political globalization increase obesity, with political globalization having the largest effect.

This study builds on this previous research by introducing several additions that foster greater confidence in the results. First, the paper lays out an explicit theoretical framework and tests additional variables not included in previous analyses that may influence global obesity trends including women’s empowerment, measures of more proximal dietary and physical activity mediating mechanisms and allows for a curvilinear relationship between GDP and BMI. Second, the paper includes a wider set of countries by employing multiple imputation to avoid selection bias and employs fixed effects to remove unobserved factors that differ between countries and are constant over time.

## Data and methods

### Dependent variable

#### Mean body mass index (BMI)

Country-level mean, age standardized BMI for men and women was accessed from the Global Burden of Metabolic Risk Factors of Chronic Diseases Collaborating Group at Imperial College London [45]. The Global Burden of Metabolic Risk Factors of Chronic Diseases measures are compiled from various sources, mostly comprised of household surveys, like the Demographic and Health Surveys based on biomarkers of height and weight that are then estimated from a Bayesian hierarchical model to provide accurate estimates across country-years. Standardized information on BMI is available for about 200 countries between 1980 and 2008 [9, 19]. Data on BMI are reported separately for men and women. However, in addition to retaining gender disaggregated estimates, we averaged male and female BMI to produce an estimate of the overall BMI in a country.

### Independent variables

#### Economic globalization

Economic globalization was measured using the Konjunkturforschungsstelle (KOF) index of economic globalization, an indicator developed by the Swiss Economic Institute that has been used in previous studies of the effect of globalization on health [10, 11, 15, 24]. Economic globalization represents a composite of two main dimensions: trade and capital flows and restrictions on trade and capital. Together this index is more comprehensive than measures that solely examine overall trade flows as it also includes barriers to trade. As globalization is a complex and multifaceted concept, we believe this measure more comprehensively captures the various dimensions of globalization that might contribute to this process leading to market penetration by multinational food corporations. Additionally, the use of an index can minimize problems of multicollinearity that arise when including multiple sub-dimensions of the same construct in a single regression. Index scores are normalized to range from 0 to 100 with higher numbers representing greater global integration. More information on this variable and other independent variables are available in Table 1. We ran all models with the overall index and with each sub-index separately and received comparable results.

**Table 1:** Descriptive statistics, imputed data

Variable	Number of obs.	Mean	SD	Minimum	Maximum
Year	5152	1994	8	1980	2008
Country Average BMI	5152	24.2	2.4	18.8	34.5
Male Average BMI	5152	23.8	2.3	19.0	33.9
Female Average BMI	5152	24.5	2.6	18.5	35.0
V-Dem Women’s Empowerment Index	5152	0.61	0.20	0	0.96
Economic Globalization	5152	51.6	17.4	9.7	98.9
Cultural Globalization	5152	40.5	21.2	5.7	93.3
GDP per capita (2005 US$)	5152	8736	11,936	100	123,263
% Urban	5152	50.1	23.8	4.3	100
Democracy (Polity IV)	5152	1.48	6.7	−10	10
Calorie Supply (kcal/capita/day)	5152	2631	481.3	1435	4085
Fat Supply (g/capita/day)	5152	75.4	33.7	13.2	175.4
Protein Supply (g/capita/day)	5152	72.9	19.9	29.4	138.1
CO2 emissions (Tons per Capita)	5152	4.3	6.46	0	68.63

#### Cultural globalization

The KOF index also captures cultural globalization through three principles categories- personal contacts, information flows and cultural proximity. We were particularly interested in the cultural proximity measures, which includes the number of McDonald’s restaurants per capita as well as the number of Ikea per capita and trade in books. As with the economic globalization measures, we ran all models with the overall index and with each sub-index separately.

#### Economic development: *GDP* per capita*, Purchasing Power Parity (PPP)*

Standard measures of gross domestic product per capita PPP were available from the Quality of Governance (QoG) indicators, which has compiled time-series data of over 2000 variables between 1980 and 2015 from the World Bank’s World Development Indicators [71]. We use GDP per capita PPP to capture the degree to which countries are developed economically, which modernization theory suggests should facilitate nutrition transitions from staple whole grains to a diet richer in energy-dense foods [51]. We use GDP per capita PPP because purchasing power parity adjusts for the cost of living in countries. This measure therefore accounts for the fact that food may be more expensive in some countries than others. We also test for a curvilinear relationship between development and BMI using a squared measure of GDP-per capita.

### Effect mediators

#### Women’s political empowerment index

The women’s political empowerment index (V-Dem) provides information on women’s civil liberties, civil society participation, and political participation spanning 190 countries from 1900 to 2012 [69]. We included this index because it had the least missing data of available measures and correlates highly with other women’s empowerment measures. As a robustness check, we also tested all models with a direct measure of women’s labor force participation, which is only available from 1990 onwards, and with different measures of women’s social and economic empowerment, including female education, that span a shorter time interval (see “Robustness Checks” in Supplementary Materials).

#### Democratization

Because countries that are democratic tend to have higher GDPs and may be more likely to have open economies and more protections for women’s rights, we control for a countries level of political democracy with the Polity IV data [40]. Polity IV is one of the most dominant data sets that political science has used to measure the level of democracy in a country. The various manuals and descriptions have been cited over 4000 times. The Polity Score captures this regime authority spectrum on a 21-pont scale ranging from − 10 (hereditary monarchy) to + 10 (consolidated democracy) and measures degree of democratic governance along six component measures including manner of executive recruitment, constraints on executive authority and political competition.

#### Urbanization

Countries have urbanized rapidly over the last several decades and urbanization is viewed as a key underlying driver of obesity trends as previously discussed [7, 50, 51, 63].

#### Food supply and carbon dioxide emissions

Research suggests that weight is ultimately a function of a lack of energy balance, or taking in more calories than are expended through physical activity [12]. We collected data from the Food and Agriculture Organization of the United Nations (FAO) on countries’ consumption of total calorie, fat and protein per capita per day as well as carbon emissions from the World Development Indicators. We use these measures to proxy for the degree to which a country has made the nutrition transition to a high protein, high fat diet. We use carbon emissions as a stand in for increasing reliance on motor vehicles as a mode of transportation, which contributes to less energy expenditure. We add these variables primarily as effect mediators to see if the effects of the main explanatory variables (women’s empowerment, economic/cultural globalization and economic development) on obesity operate through these measures.

### Analysis

#### Treatment of missing data

Although all variables spanned the full time period, 1980–2010, there were missing values in certain country-years. All countries that had at least some data available for each variable were retained in the analysis and missing data were treated as missing at random. Multiple imputation was conducted using the Amelia II software in R, a program designed to impute missing data in time-series-cross-sectional data such as this [31]. Missing data were not imputed for certain countries that were not yet established in the 1980s (i.e., the former Soviet Republics and certain Eastern European countries). All variables included in the final model were used in the imputation. Without imputation, 2435 country years of data were available. The final dataset was comprised of 5152 country-year observations. Research suggests that the listwise deletion approach in cross-national studies can lead to systematic selection bias [23]. The Additional file compares the results of models run with the imputed data with the un-imputed data.

#### Analysis approach

We ran all models as linear mixed models with country and time fixed effects to account for the clustering of data and to tease out change in the independent and dependent variables over time from stable characteristics of countries. We first ran bivariate models with each variable individually to examine their independent effects on BMI (see Table 1). Final models incorporate a five-year time lag for primary independent variables to ensure adequate time for these long run processes to influence dependent variables. However, the mediating nutritional and physical activity mechanisms were entered with two-year time lags to account for their more proximal effect on outcomes. We ran models with and without the more proximal mediating variables and we also tried one- and two-year lags were tried for each type of variable and staggered introduction of the mediating variables (see *Robustness Checks* section in Supplementary Materials).

Finally, we ran models disaggregated by high income countries (HIC) (*N* = 1469 country-years) and Low and Middle-Income Countries (*N* = 3303 country-years) to assess whether the effects of the main explanatory variables were different in these two groups of nations. We anticipated that GDP per capita to matter more in LMICs where nutrition transitions are currently underway versus HICs with more entrenched dietary practices, but we expected that economic globalization and women’s labor force participation could contribute to obesity in either setting.

We further test for a curvilinear relationship between GDP and BMI by adding GDP squared to models with all countries.

We checked for multicollinearity in two ways. First, we examined the variance inflation factors (VIFs) of each variable included in the model and excluded certain variables with excessively high VIFs (for instance, calorie supply). In addition, with retained variables, we ran the models in a stepwise fashion with each set of main independent variable to assess whether there were substantial swings in effects from adding different variables and found the models to be quite stable (see Additional file 1).

We hypothesized that the relationship between economic development and BMI would likely form a quadratic relationship with development with weight initially increasing and then leveling off over with more development. Apart from that, we were uncertain which variables- those representing dependency or modernization constructs- would prove to have more predictive power.

## Results

### Main results

Table 1 summarizes the descriptive statistics of the imputed sample and Table 2 shows bivariate regression coefficients and *p*-values for each measure included in the final models. In bivariate models, GDP-squared (though not GDP) and associated modernization measures (women’s empowerment, urbanization) each positively predicted increases in mean country BMI as did each of the mediating variables, though democratization formed a negative relationship with BMI. Neither economic nor cultural globalization was associated with increases in BMI in bivariate regressions (Table 2).

**Table 2:** Bivariate regression results for each predictor with time and country fixed effects^a^

Variable	Coefficient	p-value	Model R-Square
V-Dem Women’s Empowerment Index, 5 yr. lag	0.74	0.006	0.09
GDP per capita PPP (1000 2005 US$), 5 yr. lag	0.0008	0.555	0.08
GDP per capita PPP Squared (1000 2005 US$), 5 yr. lag	0.005	0.000	0.15
Economic Globalization, 5 yr. lag	0.00	0.449	0.07
Cultural Globalization, 5 yr. lag	0.00	0.456	0.09
Democracy (Polity IV), 5 yr. lag	− 0.02	0.000	0.06
% Urban, 5 yr. lag	0.02	0.000	0.32
Calorie Supply (kcal/capita/day), 2 yr. lag	0.00	0.000	0.24
Fat Supply (g/capita/day), 2 yr. lag	0.01	0.000	0.21
Protein Supply (g/capita/day), 2 yr. lag	0.02	0.000	0.25
Carbon Dioxide Emissions (Tons per Capita), 2 yr. lag	0.04	0.000	0.17

^a^Models include time and country fixed effects but no other covariates. Dependent variable is the average BMI across all countries

In the multivariate models with all countries (Table 3, Models 1–3), trends from the bivariate models persisted however with some different effects for men and women. Women’s BMI decreased with GDP whereas men’s BMI increased. Cultural globalization decreased women’s BMI, but increased men’s. Otherwise results were consistent between female and male BMI. Women’s empowerment predicted a 0.55 kg/m2 increase in BMI per unit change in independent variable (moving from 0 to 1) adjusting for all other factors and held across both men and women. Economic globalization was not associated with BMI across models (Table 3, Models 1–3). The addition of mediating variables (representing the food and physical activity environment) did not fully explain the effect of the modernization-related variables on BMI, however, each variable contributed to higher mean BMIs with the exception of total fat consumption (Table 3, and also Additional file for stepwise model). Urbanization predicted higher mean BMIs while democracy was associated with lower BMIs over time.

**Table 3:** Multiple regression fixed effects models

	All countries			LMICs			HICs		
	Total	Female	Male	Total	Female	Male	Total	Female	Male
	(Coef, CI)	(Coef, CI)	(Coef, CI)	(Coef, CI)	(Coef, CI)	(Coef, CI)	(Coef, CI)	(Coef, CI)	(Coef, CI)
Real GDP per Capita (1000 2005US$), PPP 5 yr. lag	− 0.0049***	− 0.0132***	0.0035**	0.0477***	0.0323***	0.0631***	−0.0065***	− 0.0092***	− 0.0038***
	[− 0.0072,-0.0025]	[− 0.0162,-0.0102]	[0.0013,0.0056]	[0.0356,0.0598]	[0.0172,0.0475]	[0.0520,0.0743]	[−0.0090,-0.0040]	[−0.0125,-0.0059]	[− 0.0057,-0.0019]
Economic Globalization, 5 yr. lag	− 0.1007	−0.1378	− 0.0635	− 0.0276	− 0.027	− 0.0282	− 0.4988**	− 0.5568*	− 0.4407***
	[− 0.2855,0.0842]	[− 0.3741,0.0984]	[− 0.2288,0.1018]	[− 0.2474,0.1922]	[− 0.3019,0.2479]	[− 0.2300,0.1737]	[−0.8224,-0.1751]	[− 0.9863,-0.1272]	[− 0.6892,-0.1922]
Cultural Globalization, 5 yr. lag	0.0846	−1.0025***	1.1717***	0.215	−0.5755**	1.0056***	−0.5068**	−1.1211***	0.1074
	[−0.1219,0.2910]	[−1.2663,-0.7388]	[0.9871,1.3562]	[−0.0895,0.5195]	[−0.9563,-0.1947]	[0.7260,1.2852]	[−0.8291,-0.1846]	[−1.5489,-0.6933]	[− 0.1400,0.3548]
Women’s Empower-ment index, 5 yr. lag	0.5548***	0.5370***	0.5727***	0.4898***	0.3134**	0.6662***	1.2394***	1.7150***	0.7638***
	[0.3894,0.7203]	[0.3255,0.7484]	[0.4248,0.7207]	[0.3076,0.6720]	[0.0856,0.5413]	[0.4989,0.8335]	[0.8624,1.6164]	[1.2146,2.2155]	[0.4743,1.0533]
Democracy, 5 yr. lag	−0.0165***	−0.0147***	−0.0182***	− 0.0145***	−0.0143***	− 0.0146***	−0.0109**	− 0.0102*	−0.0116***
	[−0.0194,-0.0136]	[−0.0185,-0.0110]	[− 0.0209,-0.0156]	[− 0.0177,-0.0112]	[−0.0184,-0.0103]	[− 0.0176,-0.0116]	[− 0.0178,-0.0040]	[−0.0193,-0.0010]	[− 0.0169,-0.0063]
Urban Population (%), 5 yr. lag	0.0197***	0.0243***	0.0151***	0.0197***	0.0210***	0.0185***	0.0110**	0.0108*	0.0112***
	[0.0162,0.0232]	[0.0198,0.0288]	[0.0119,0.0182]	[0.0157,0.0238]	[0.0159,0.0261]	[0.0147,0.0222]	[0.0039,0.0181]	[0.0014,0.0203]	[0.0057,0.0167]
Fat supply (g/capita/day), 2 yr. lag	−0.2298	−0.5855	0.1259	0.0601	−0.2523	0.3725	−0.4245	−1.2499*	0.4009
	[−0.7781,0.3185]	[−1.2861,0.1152]	[−0.3645,0.6162]	[− 0.6256,0.7458]	[− 1.1098,0.6052]	[− 0.2572,1.0021]	[−1.3135,0.4645]	[−2.4299,-0.0699]	[−0.2817,1.0834]
Protein supply (g/capita/day), 2 yr. lag	5.2932***	6.3267***	4.2597***	5.5366***	6.5930***	4.4803***	3.7067***	4.5127***	2.9006***
	[4.5661,6.0203]	[5.3976,7.2557]	[3.6095,4.9099]	[4.6805,6.3927]	[5.5223,7.6636]	[3.6942,5.2664]	[2.3864,5.0270]	[2.7602,6.2651]	[1.8869,3.9143]
Carbon Dioxide Emissions (Tons per Capita), 2 yr. lag	0.0246***	0.0231***	0.0262***	0.0821***	0.1053***	0.0588***	0.0206***	0.0232***	0.0181***
	[0.0189,0.0304]	[0.0158,0.0304]	[0.0211,0.0313]	[0.0640,0.1002]	[0.0827,0.1279]	[0.0422,0.0755]	[0.0146,0.0267]	[0.0151,0.0312]	[0.0134,0.0227]
_cons	21.2243***	21.5672***	20.8815***	20.6110***	21.0407***	20.1813***	23.0198***	23.1230***	22.9165***
	[21.014,21.434]	[21.298,21.835]	[20.693,21.069]	[20.409,20.812]	[20.788,21.292]	[19.996,20.366]	[22.447,23.592]	[22.363,23.882]	[22.477,23.355]
r2	0.8217	0.7798	0.8209	0.8317	0.8167	0.7974	0.8346	0.721	0.9054
N	4206	4206	4206	2911	2911	2911	1295	1295	1295

*time fixed effects entered but not shown. Significance level: * *p* < .05, ** for *p* < .01, and *** for *p* < .001

### Results disaggregated by high-income and low- and middle-income countries

Table 3 **(**models 4–9) summarize the results disaggregated by high-income and low- and middle-income countries (and further disaggregated by male and female BMI). Across the models, women’s empowerment is positively associated with increasing BMI in both higher and lower income countries. However, the effect of women’s empowerment was greater in high-income countries. In high income countries, a one unit increase in women’s empowerment is associated with 1.24 kg/m2 increase in BMI over time and 0.49 kg/m2 increase in BMI in LMICs (Table 3 Models 7 & 4 respectively).

There were other differential trends in effects of other independent variables in high-income and low and middle-income countries. Increasing GDP per capita predicted higher BMIs in LMICs, but lower BMIs in HICs consistent with the idea of a curvilinear relationship between GDP and BMI. Specifically, in LMICs, a $1000 increase in GDP per capita produced a 0.05 kg/m2 increase in BMI over time. Even disaggregated by high- and low-income countries, democracy negatively predicted BMI. Economic globalization had no effect on weight in LMICs but had a negative effect on male BMI in HICs (increases in economic globalization predicted lower BMIs in men in HICs). Cultural globalization was associated with higher BMI in men in LMICS and lower BMI in women in both LMICs and HICs.

### Quadratic relationship

**Fig. 2:**
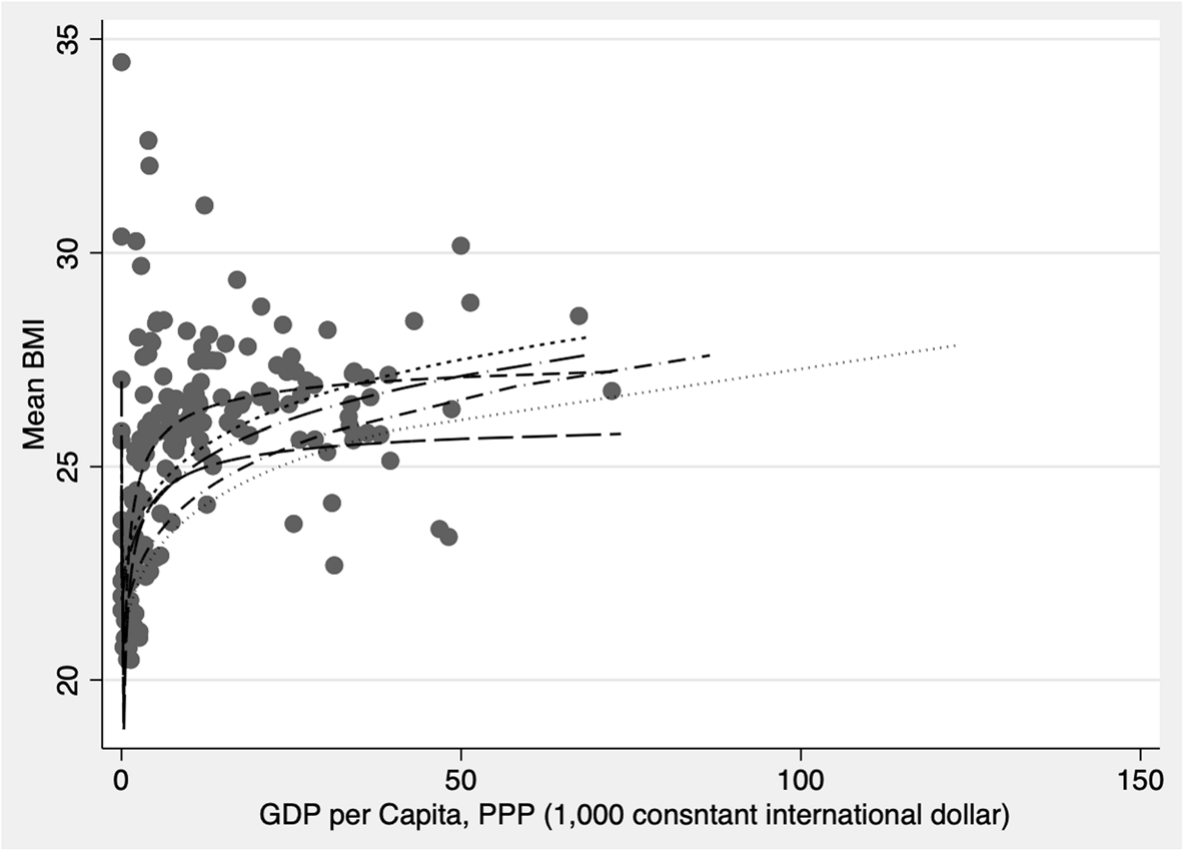
Quadratic Plots of the Relationship between GDP and BMI, 1980–2008

**Fig. 3:**
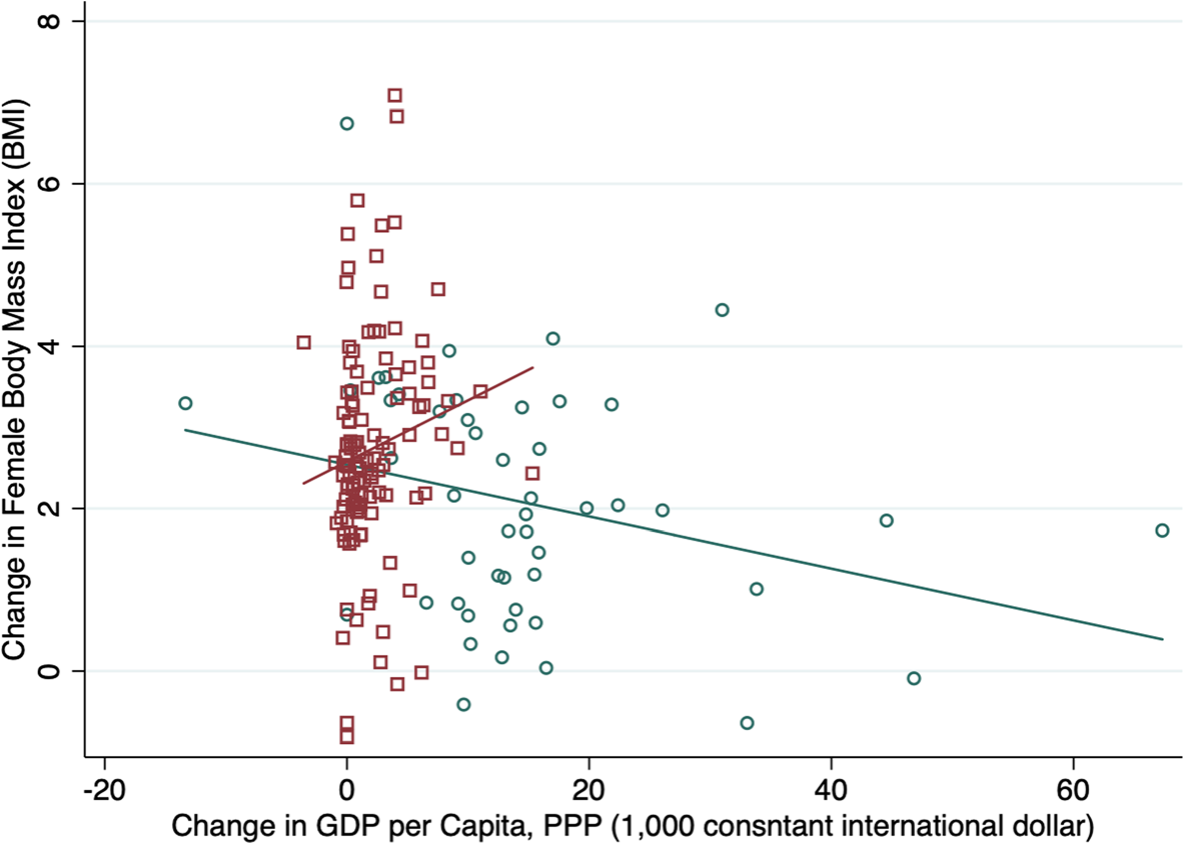
Change in Female BMI by Change in GDP per capita (1980–2008)

**Fig. 4:**
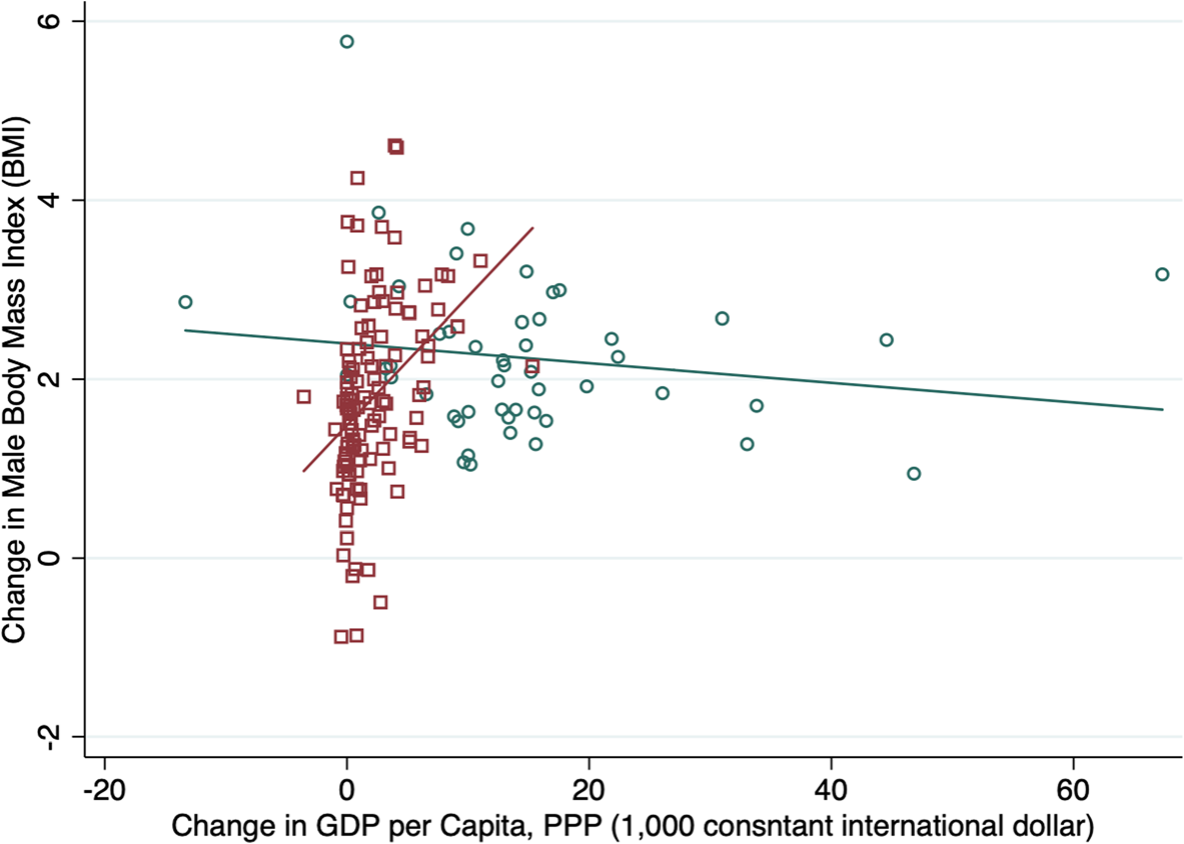
Change in Male BMI by Change in GDP per capita (1980–2008)

Table 4 show the relationship between obesity and a quadratic transformation of GDP (GDP squared). In keeping with expectation, while the linear term shows a positive relationship between BMI and GDP, GDP-squared shows a negative relationship. This suggests that there is a hump-shape relationship between GDP and weight where at low levels of development increasing GDP is associated with higher weight, but this relationship flattens at higher levels of GDP. However, this relationship appears to primarily hold for male weight whereas for women, more GDP is more consistently associated with lower weight. Graphic representation of the relationship between GDP and BMI shows more of an inverted J-shape (ore fractional polynomial) relationship between income per capita and weight with a number of lower income Pacific Island countries with very high BMIs falling above the trend line (see Fig. 2 and Additional file 1: Figures S1-S4). We also graph the relationship between the change in GDP and change in BMI over the 30-year period and fit plots for low/middle and high-income countries separately. We find that whereas increases in GDP over the period are strongly linearly associated with increases in BMI in LMICs, in HICs, increases in GDP are negatively associated with BMI (see Figs. 3 and 4).

**Table 4:** Multiple regression fixed effects models with GDP-squared, all countries

	All countries		
	Total	Female	Male
	(Coef, CI)	(Coef, CI)	(Coef, CI)
Real GDP per capita (1000 2005US$), PPP 5 yr. lag	0.0022	−0.0115***	0.0159***
	[−0.0020,0.0063]	[−0.0169,-0.0062]	[0.0122,0.0196]
GDP pc PPP-Squared	−0.0001***	− 0.00001	−0.0002***
	[−0.0001,-0.0001]	[−0.0001,0.0000]	[− 0.0002,-0.0001]
Economic Globalization, 5 yr. lag	− 0.0676	−0.13	− 0.0052
	[−0.2528,0.1176]	[− 0.3671,0.1071]	[− 0.1699,0.1594]
Cultural Globalization, 5 yr. lag	0.0062	−1.0211***	1.0335***
	[−0.2032,0.2157]	[−1.2893,-0.7529]	[0.8473,1.2198]
Women political empowerment index, 5 yr. lag	0.5638***	0.5391***	0.5885***
	[0.3986,0.7290]	[0.3276,0.7506]	[0.4416,0.7353]
Democracy, 5 yr. lag	−0.0163***	− 0.0147***	−0.0179***
	[−0.0192,-0.0134]	[−0.0184,-0.0110]	[− 0.0205,-0.0153]
Urban Population (%), 5 yr. lag	0.0203***	0.0245***	0.0161***
	[0.0168,0.0238]	[0.0200,0.0290]	[0.0130,0.0193]
Fat supply (g/capita/day), 2 yr. lag	−0.1775	− 0.5731	0.2181
	[−0.7253,0.3704]	[−1.2745,0.1283]	[−0.2689,0.7052]
Protein supply (g/capita/day), 2 yr. lag	5.2939***	6.3268***	4.2610***
	[4.5682,6.0196]	[5.3978,7.2559]	[3.6159,4.9061]
Carbon Dioxide Emissions (Tons per Capita), 2 yr. lag	0.0249***	0.0232***	0.0266***
	[0.0192,0.0306]	[0.0158,0.0305]	[0.0215,0.0317]
_cons	21.1671***	21.5536***	20.7805***
	[20.9555,21.3786]	[21.2827,21.8245]	[20.5924,20.9686]
r2	0.8224	0.7798	0.8237
N	4206	4206	4206

*time fixed effects entered but not shown. Significance level: * p < .05, ** for p < .01, and *** for p < .001

#### Robustness checks

The core findings outlined above were quite robust to different model specifications and analysis approaches yielding greater confidence in the results. The Statistical Additional file includes several additional model specifications including models introduced in a stepwise fashion to see the impact of the inclusion of different variables into the model on other coefficients; models with no time lags; models with the unimputed data; and models with alternative measures of women’s empowerment (e.g., female labor force participation; an alternative measure of women’s social empowerment; women’s educational attainment). The main results that were different across these models was that in the models with no time lags and unimputed data, we found a negative relationship between economic globalization and mean BMI, including in LMICs, especially in the unimputed models. All three additional measures of women’s empowerment exhibited a consistently positive relationship with BMI across countries, except for women’s educational attainment, which showed a negative relationship with BMI in HICs, but a positive relationship in LMICs.

## Discussion

We tested two theories related to the relationship between economic development and health, which we have broadly termed the “dependency/world systems theory view” and the “modernization view.” In the dependency/world systems theory view, rising obesity rates across countries over time can be attributed primarily to external structural forces, particularly Big Food and harmful trade deals that flood countries with obesogenic, nutrient-poor foods leading to increasing weight gain and associated chronic conditions. By contrast, the modernization theory view sees countries as progressing through a series of teleological stages as they develop economically that lead inexorably to nutrition transitions from lower calorie, primarily plant-based diet to a meat and processed food diet associated with weight gain and chronic illness. This, and associated processes, including women’s rights and democratization may lead to weight gain as countries become more stable, affluent and egalitarian.

Using a large cross-national longitudinal dataset, we found that in spite of the broad literature suggesting negative impacts of trade liberalization on obesity (e.g., [10, 17, 18, 28, 29, 37, 64, 65]), economic globalization has had no discernable effect on mean BMI in countries over time. Moreover, in high income countries, the models suggested that economic globalization may be associated with *lower* mean BMI. This result was robust to multiple model specifications and is consistent with results from prior studies [10, 11, 24, 35]. In fact, consistent with prior studies, in models with unimputed data, we found evidence of a consistent negative relationship between economic globalization and BMI including in LMICs [10, 11, 24, 35].

Cultural globalization was also not significant overall, but appeared to have different effects for men and women and in higher income and lower income settings. Cultural globalization was associated with higher mean BMI for men in LMICs but lower mean BMI in women in HICs and LMICs. Although we were hypothesizing that countries that are more Westernized might have adopted a stronger fast-food/processed food culture (i.e., through McDonaldization or coca-colalization processes) leading to higher body weights, we found that cultural globalization was associated with lower body weights in women. Among women it is possible that cultural globalization may influence conventions on standards of beauty, including idealized notions of low female body weight that contribute to women dieting to achieve a slimmer body size [11].

By contrast, we largely find support for the “modernization” view that low- and middle- income countries are experiencing widespread nutrition transitions as they develop economically. Among LMICs, GDP per capita PPP positively predicted BMI. Among HICs, there was a negative relationship between GDP and BMI over time. This supported our theory that obesity would operate more as a disease of affluence than a disease of poverty whereby past a certain level of development, more income would improve health by lowering BMI, much like the relationship between life-expectancy and health [74]. The hump-shape relationship supports the view that middle-income countries are especially susceptible to obesity and counters the view that low-income countries are experiencing a double disease burden to the same degree as middle-income countries. While some have suggested that in parallel with trends in developed countries, it is increasingly the poor in developing countries that suffer from obesity [65, 76], other studies examining social gradients in obesity within countries have refuted this claim. For instance, Subramanian et al 2011 [68], found that in low income countries, the overweight burden was mostly concentrated among higher SES individuals, and that the wealth gradient became less marked only at a level of per capita GDP of about 5500 USD. Monteiro et al 2004 [44], found the reversal in the obesity gradient occurred at a level of GNP per capita of about 2500 US dollars, which was broadly supported by a recent systematic review [13] and a large microstudy of over 244 Demographic and Health Surveys across 56 countries [25]. Together these results suggest greater support for a modernization view in which health transitions occur in a relatively staged and predictable manner.

However, we also found some exceptions to the hump-shaped trend. The disproportionately high BMIs and increases in BMI observed in small Pacific Island countries have been previously identified but warrant further discussion as their experience diverged from overall trends. Small island nations are thought to be particularly susceptible to weight gain and chronic illnesses for a variety of reasons related to their geography as well as epi-genetics. The necessity of importation of foodstuffs as well as colonial ties and incorporation into global trade regimes have contributed to changing availability and accessibility of processed imported foods over the last 30 years in small island nations, which may interact with genetic substrates to contribute to weight gain and chronic diseases in these contexts [33, 43, 62]. On the genetic side, the thrifty genotype hypothesis postulates that obesity and type 2 diabetes are caused by positive selection of genotypes for efficiency of metabolism and energy and fat storage, thereby conferring advantage in times of nutrient scarcity. This hypothesis has been widely used to explain the extraordinarily high rates of diabetes seen among Pima Indians and other indigenous populations [33]. It has been suggested that these populations may have an enhanced genetic predisposition to obesity and diabetes because of overrepresentation of the thrifty genotypes, resulting from evolutionary selection by repeated feast and famine cycles, which, interacting with the food environment, is speculated to also be the case for Pacific Island groups.

We found women’s empowerment to be among the strongest and most consistent predictor of increases in mean BMI in countries over time. Overall, countries that increased by one unit in women’s empowerment each year, increased their BMI by 0.55 kg/m2. This is a significant amount given that the mean change in BMI across all countries was 2.4 kg/m2 over the last 30 years. Substantively, holding all other variables at their mean values, raising women’s empowerment by 1 unit would raise global BMI by 0.12 points, which is roughly 2% of a standard deviation of the dependent variable. The results were even stronger among high-income countries where a one unit increase in women’s empowerment is associated with 1.66 kg/m2 increase in BMI over time among women and 0.74 kg/m2 for men. We found similar results (positive and significant relationship) for other measures of women’s empowerment, including women’s labor force participation, women’s social rights and women’s education for 1990+ (available in Additional file 1). These results were unexpected, robust and quite novel as previous research has not to our knowledge examined the association between changes in women’s rights and the effect on weight at a country level. These macro-level findings across countries parallel findings from individual-level research that shows that children of women who work are more likely to be overweight and obese [1, 3, 5, 26]. Micro-level research may consider studying these effects more closely.

While these results regarding the impact of women’s empowerment on BMI are novel, they must be interpreted with caution. As we are using fixed effects, the results are best understood as the effect of *change* in the independent variables on the *change* in the dependent variable- countries in which women’s empowerment has increased have seen higher growth in mean BMI. While this is a good approach to measure accurately the temporal sequence of events, this should not be interpreted as the same as saying that countries with more women’s empowerment have higher BMIs. For instance, Middle East and North African countries have among the highest BMIs internationally among women and receive consistently low women’s empowerment scores.

Urbanization also predicted higher mean BMI. We take these as additional markers in support of the modernization theory view that internal economic development processes more so than global trade regimes are contributing to rising body size internationally.

However, the finding of a negative relationship between democratic governance and mean BMI is surprising given research that has suggested that democracies are better able to avoid famines suggesting that democratic governance should be associated with higher mean BMI at least at lower income levels [61]. Yet, we found that even among low- and middle-income countries, democracy was associated with lower BMI. It may be that more established democracies are better able to adopt public health policies such as regulations on marketing of products that protect the public against the influence of big food.

In both high and low-income countries, increases in protein intake and carbon emissions predicted higher mean BMI as did increases in protein, but not consistently fat supply. Yet, introducing measures designed to capture the degree to which a country has gone through a nutrition transition (e.g., amount of fat, protein, and carbon emissions) in a stepwise fashion did not eliminate the effect of GDP or women’s empowerment suggesting additional pathways contribute to obesity via economic development not captured here (see Additional file 1).

### Implications

Our findings suggest that the health harms from global trade regimes may be overstated and that studies asserting the importance of these external obesity diffusion mechanisms should also account for domestic social transformations that may be contributing to weight gain. Much of the weight gain within countries over the past 30 years seems to be explained by other factors including changes in women’s rights, urbanization and economic development. While Big Food is an easy scapegoat for concerns about the potential rise in chronic illness stemming from overweight/obesity, it is equally possible that overindulgence in locally grown foodstuffs coupled with changes in physical activity and gender roles are the primary drivers of weight gain. However, although we did not ultimately find a relationship between markers of economic globalization and cross-national increases in BMI, this does not imply that these factors have no consequence. Finally, childhood obesity may be more important to assess than increases in adult body weight as life style changes are likely to be more manifest among youth rather than among the current adult population, although de Soysa & de Soysa (2017) [11] find that economic globalization has a negative relationship with childhood BMI.

#### Limitations

The results from this analysis must be interpreted with some caution. For one, the variables used to capture the broad social constructs we are measuring are crude at best. Measures of trade openness for instance, is not a precise measure of the penetration of Big Food into domestic markets. Carbon emissions are not an ideal measure of physical activity. GDP per capita does not account for the distribution of incomes within countries and data on inequality were too sparse to include. These and other measures are at best crude approximations of underlying constructs. Nevertheless, this study goes beyond other studies in trying to parse out the impact of more distal social and structural influences from more proximal behavior changes that might predict increases in body weight over time.

Furthermore, in spite of relatively good data coverage on certain variables, other variables required more imputation to ensure a balanced panel. Consequently, nearly half of the observations needed to be imputed. The unimputed models were ultimately consistent with the imputed model results and we have preferred the imputed model results since it limits countries missing data. However, we cannot know for certain if the results would be the same if original measures were available across all data points. Finally, although we have tried to adjust for multicollinearity, as with all macro-studies, each of the social processes we are testing (economic development, women’s empowerment, globalization, democratization, urbanization) are at least somewhat interrelated impeding our ability to tease out independent effects. Our models were quite stable and did not exhibit symptoms of multicollinearity in spite of elevated VIF scores on certain measures. Moreover, country-level relationships must be interpreted differently from individual-level effects. How the effects of macro-processes “trickle-down” to affect the micro-determinants of weight cannot be assessed in this analysis. We do not have a direct measure of transnational food and beverage company entry into local markets, which is the main theoretical mechanism by which globalization is believed to influence weight in LMICs. In spite of these inherent limitations in design, the strengths of this analysis lie in the systematic approach undertaken with a large number of countries, over a 30-years time period. Even recent studies employing very rich microdata lose a full global picture by necessarily restricting time and country trends.

## Conclusions

Using a longitudinal dataset of mean body mass index that covers 190 countries from 1980 to 2008 and testing several competing theories for the global rise in obesity, we find that processes associated with economic development, including women’s empowerment and urbanization, but not globalization, robustly predicts higher mean BMI in men and women in both high- and low/middle income countries. International obesity research should pay closer attention to the domestic factors that contribute to rising obesity rates internationally.

## Additional file

Additional file 1: **Table S1.** Variance Inflation Factors for all Variables in Models. **Table S2.** Stepwise Introduction of Variables with GDP-squared, All Countries. **Table S3.** Stepwise introduction of Variables with GDP-squared, Stratified by LMICs and HICs. **Table S4.** Imputed main models with no time lags. **Table S5.** Unimputed Main Models with time lags. **Table S6.** Alternative Women’s Empowerment Measures (Unimputed Main Models with Time Lags). **Table S7.** Models with Female Educational Attainment as an Alternative Measure of Women’s Empowerment. **Figure S1.** Relationship between GDP and BMI, 1980-2008. **Figure S2.** Relationship between GDP and BMI, NO LABELS, 1980-2008. **Figure S3.** Quadratic Plots of the Relationship between GDP and BMI, 1980-2008. **Figure S4.** Change in BMI by Change in GDP per capita (1980-2008). (DOCX 18668 kb)
