# Supplementary material for: What is driving global obesity trends? Globalization or “modernization”?
Source: Global Health. 2019 Apr 27;15:32. doi: 10.1186/s12992-019-0457-y (PMC6486955; doi:10.1186/s12992-019-0457-y)

**Additional file**

List of Appendices

Table S1: Variance Inflation Factors for all Variables in Models

Table S2: Stepwise Introduction of Variables with GDP-squared, All Countries

Table S3: Stepwise introduction of Variables with GDP-squared, Stratified by LMICs and HICs

Table S4: Imputed main models with no time lags

Table S5: Unimputed Main Models with time lags

Table S6: Alternative Women’s Empowerment Measures (Unimputed Main Models with Time Lags)

Table S7: Models with Female Educational Attainment as an Alternative Measure of Women’s Empowerment

Table S1: Variance Inflation Factors for all Variables in Models

| Variable | VIF | 1/VIF |
| --- | --- | --- |
| L5.dr_eg | 22.76 | 0.043934 |
| L5.dr_sg | 24.94 | 0.0401 |
| L5.wdi_gdpc | 13.64 | 0.073332 |
| L5.gdpc_square | 5.78 | 0.173051 |
| L5.wdi_urban | 11.96 | 0.083626 |
| L5.vdem_gender | 21.34 | 0.046855 |
| L5.p_polity2 | 1.87 | 0.535221 |
| L2.fat_supply | 27.75 | 0.036034 |
| L2.protein_supply | 44.42 | 0.022515 |
| L2.wdi_co2 | 3.21 | 0.311221 |
| year |  |  |
| 1986 | 1.59 | 0.629963 |
| 1987 | 1.59 | 0.626973 |
| 1988 | 1.6 | 0.625523 |
| 1989 | 1.61 | 0.62245 |
| 1990 | 1.6 | 0.623077 |
| 1991 | 1.61 | 0.622296 |
| 1992 | 1.61 | 0.621841 |
| 1993 | 1.6 | 0.623201 |
| 1994 | 1.6 | 0.623269 |
| 1995 | 1.62 | 0.617142 |
| 1996 | 1.64 | 0.609915 |
| 1997 | 1.72 | 0.582004 |
| 1998 | 1.75 | 0.571351 |
| 1999 | 1.77 | 0.565937 |
| 2000 | 1.78 | 0.561184 |
| 2001 | 1.79 | 0.55925 |
| 2002 | 1.8 | 0.555904 |
| 2003 | 1.82 | 0.550724 |
| 2004 | 1.83 | 0.547177 |
| 2005 | 1.85 | 0.53989 |
| 2006 | 1.85 | 0.540975 |
| 2007 | 1.87 | 0.535769 |
| 2008 | 1.88 | 0.532806 |
| Mean VIF | 6.58 |  |

Table S2: Stepwise Introduction of Variables with GDP-squared, All Countries


*time fixed effects entered but not shown. Significance level: * p<.05, ** for p<.01, and *** for p<.001

Table S3: Stepwise introduction of Variables with GDP-squared, Stratified by LMICs and HICs

*time fixed effects entered but not shown. Significance level: * p<.05, ** for p<.01, and *** for p<.00

Table S4: Main models with no time lags

*time fixed effects entered but not shown. Significance level: * p<.05, ** for p<.01, and *** for p<.001

Table S5: Unimputed Main Models with time lags

*time fixed effects entered but not shown. Significance level: * p<.05, ** for p<.01, and *** for p<.001

Table S6: Alternative Women’s Empowerment Measures (Unimputed Main Models with Time Lags)

*time fixed effects entered but not shown. Significance level: * p<.05, ** for p<.01, and *** for p<.001

Table S7: Models with Female Educational Attainment as an Alternative Measure of Women’s Empowerment

*time fixed effects entered but not shown. Significance level: * p<.05, ** for p<.01, and *** for p<.001

Figure S1 Graphs A1-A4: Relationship between GDP and BMI, 1980-2008


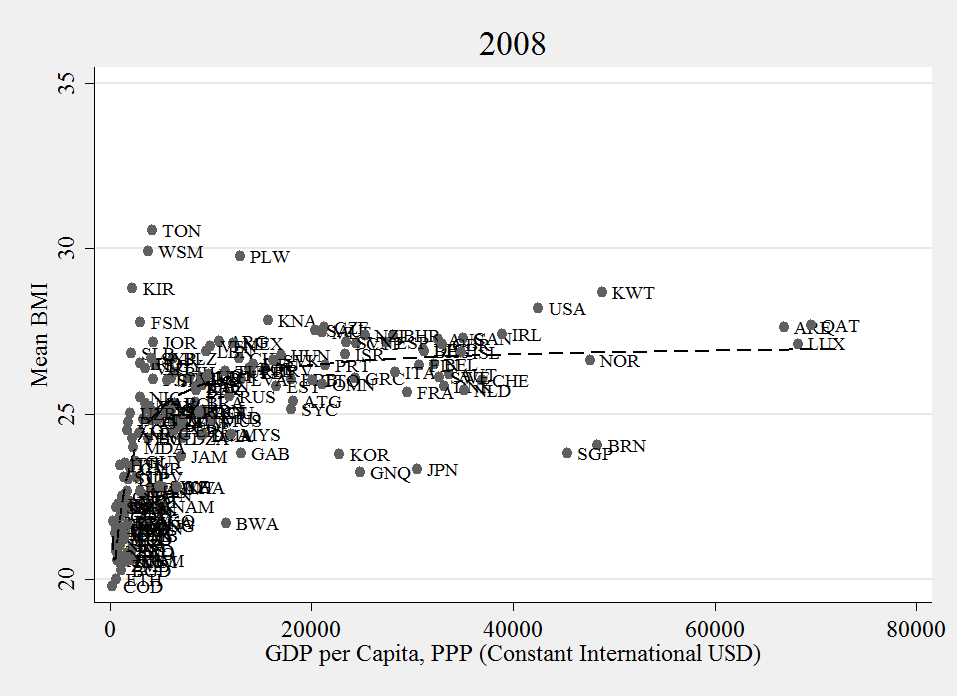

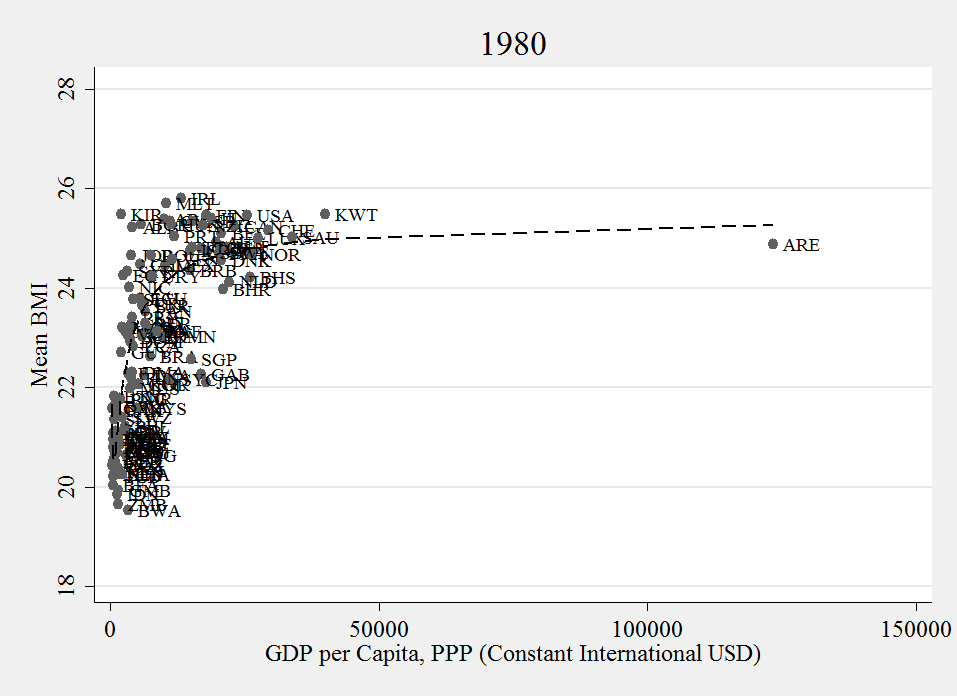

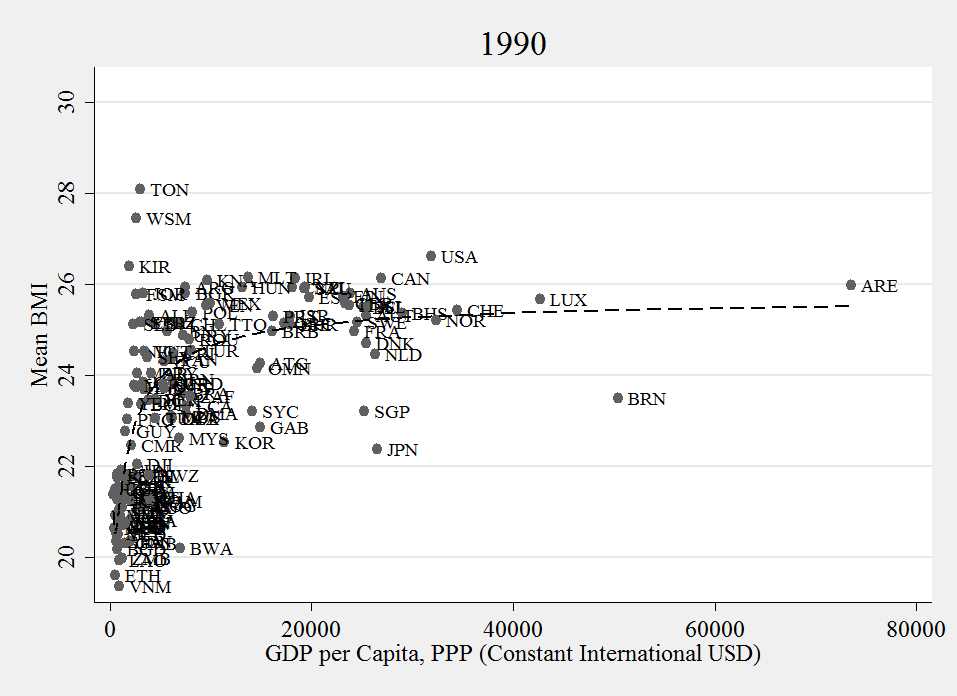

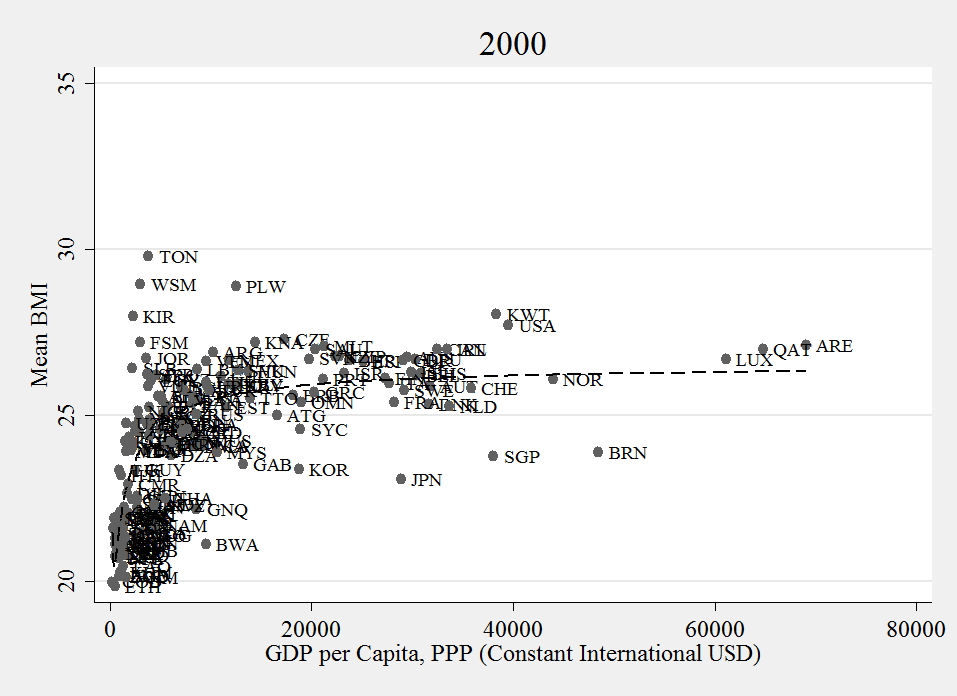


Figure S2

Graphs A1-A4: Relationship between GDP and BMI, NO LABELS, 1980-2008


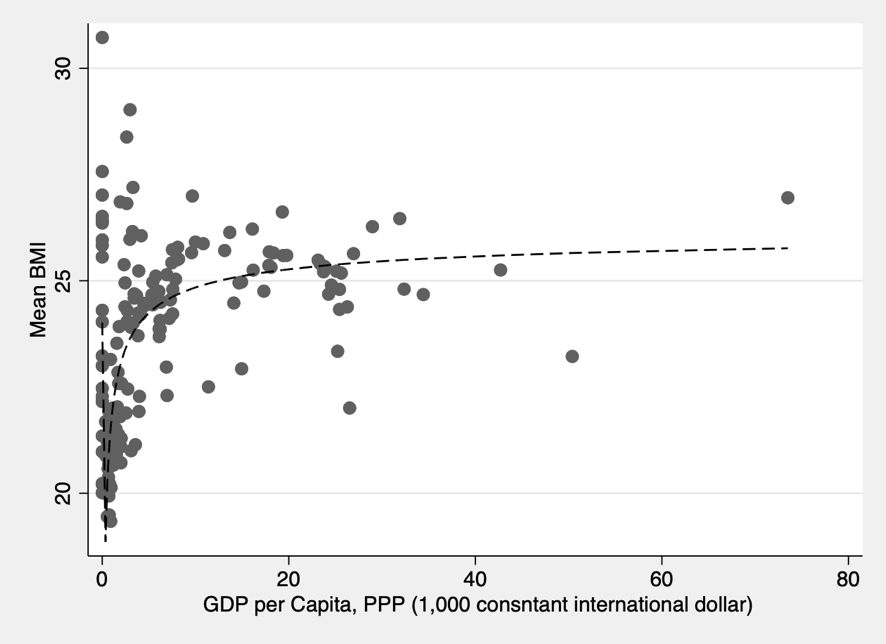

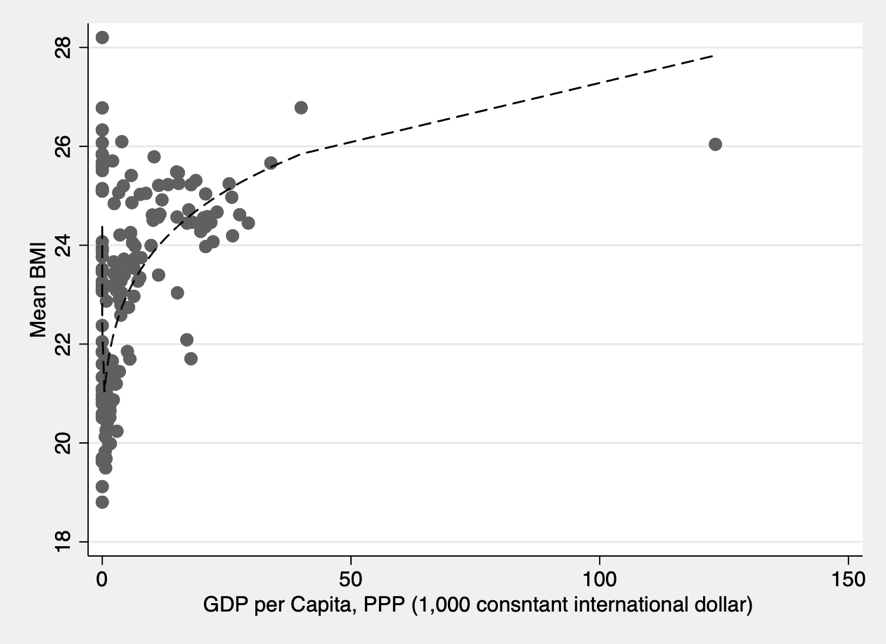


1990

1980

2008


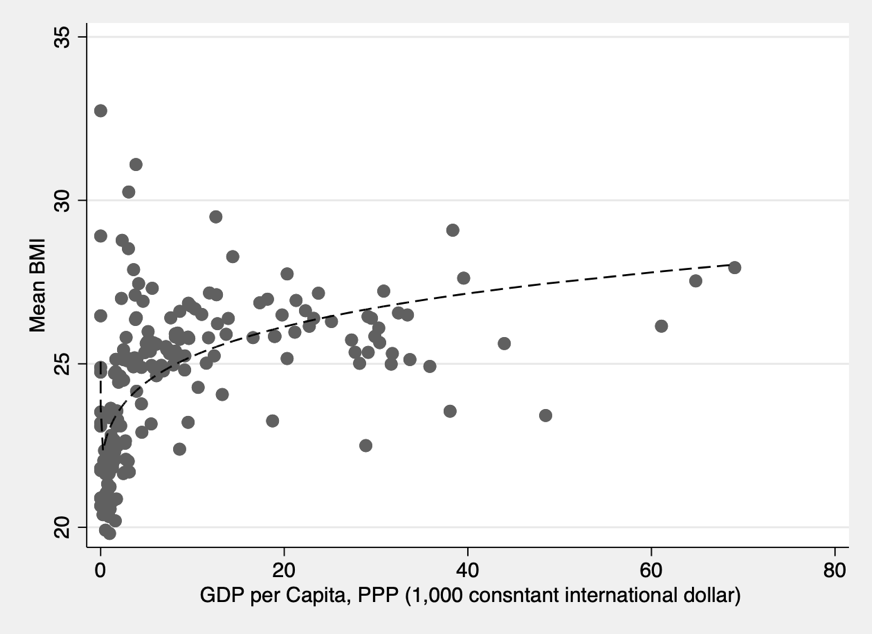

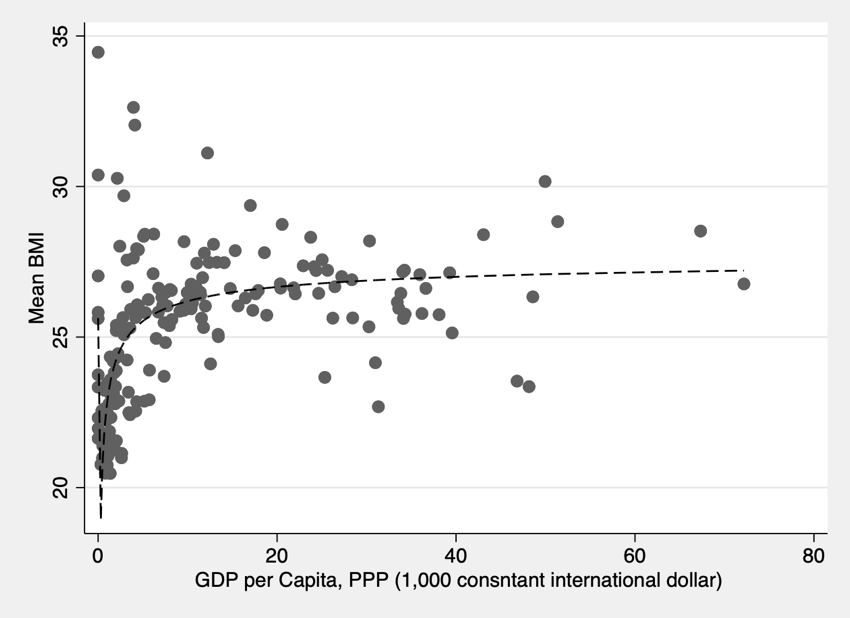


2000

Graphs from paper with no labels

Figure S3: Quadratic Plots of the Relationship between GDP and BMI, 1980-2008


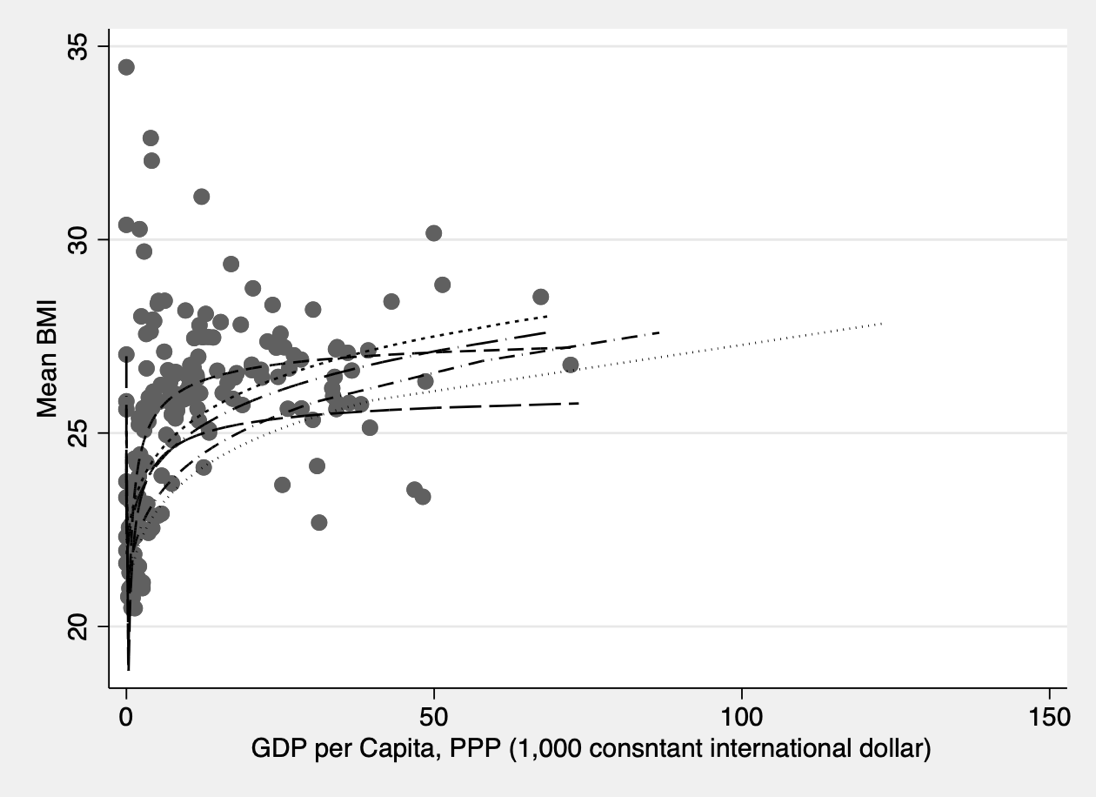


Figure S4: Change in BMI by Change in GDP per capita (1980-2008)

Men

Women


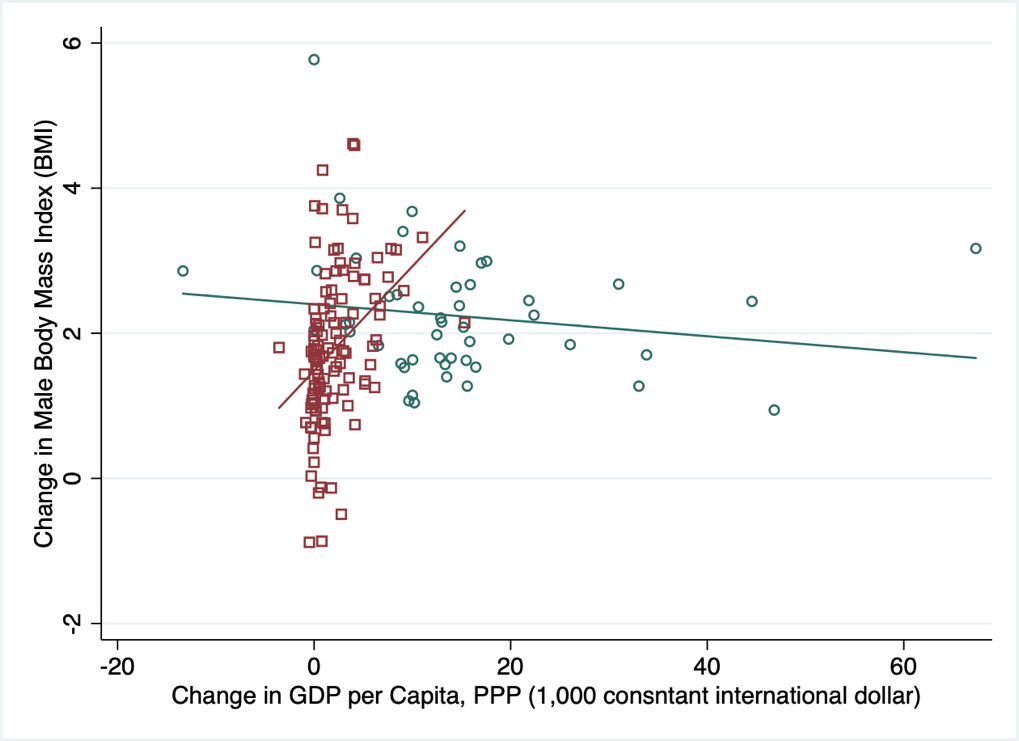

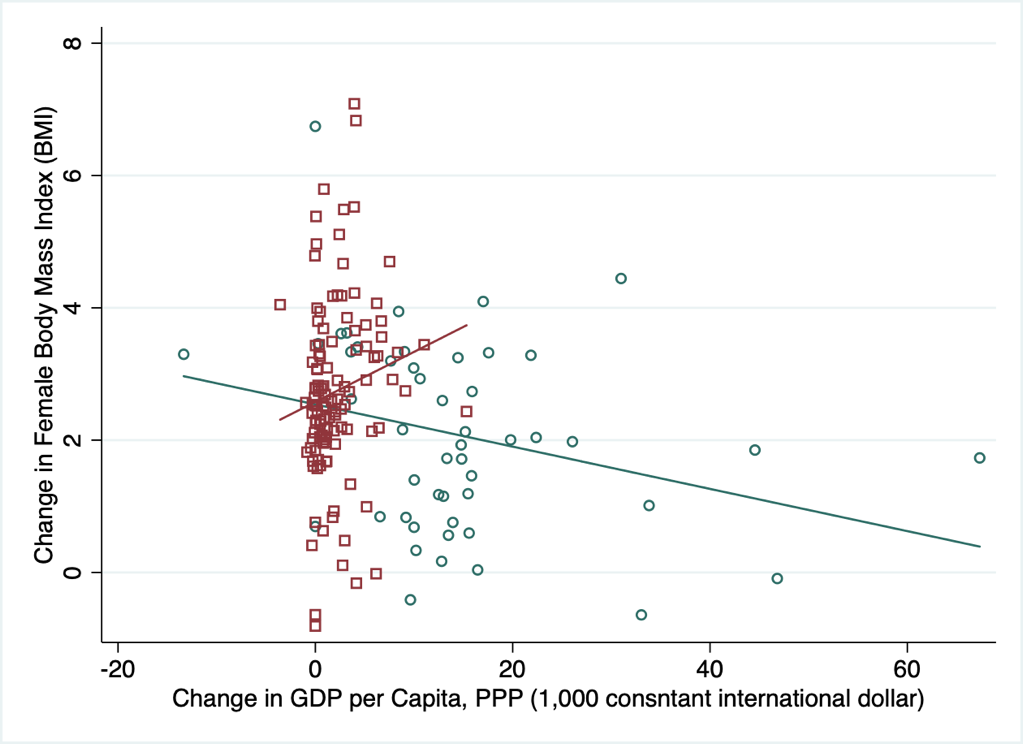

Supplement: Supplementary file 1 — Table S1. Variance Inflation Factors for all Variables in Models. Table S2. Stepwise Introduction of Variables with GDP-squared, All Countries. Table S3. Stepwise introduction of Variables with GDP-squared, Stratified by LMICs and HICs. Table S4. Imputed main models with no time lags. Table S5. Unimputed Main Models with time lags. Table S6. Alternative Women’s Empowerment Measures (Unimputed Main Models with Time Lags). Table S7. Models with Female Educational Attainment as an Alternative Measure of Women’s Empowerment. Figure S1. Relationship between GDP and BMI, 1980-2008. Figure S2. Relationship between GDP and BMI, NO LABELS, 1980-2008. Figure S3. Quadratic Plots of the Relationship between GDP and BMI, 1980-2008. Figure S4. Change in BMI by Change in GDP per capita (1980-2008). (DOCX 18668 kb) [file 12992_2019_457_MOESM1_ESM.docx]
